# The role of definitive chemoradiotherapy versus surgery as initial treatments for potentially resectable esophageal carcinoma

**DOI:** 10.1186/s12957-018-1470-y

**Published:** 2018-08-17

**Authors:** Ming-Wei Ma, Xian-Shu Gao, Xiao-Bin Gu, Mu Xie, Ming Cui, Min Zhang, Ling Liu, Huan Yin, Long-Qi Chen

**Affiliations:** 1 Department of Radiation Oncology, Peking University First Hospital, No.7 Xishiku Street, Beijing, 100034 People’s Republic of China; 2 Department of Medical and Pharmaceutical Science and Technology Strategy Research, Institute of Medical Information, Chinese Academy of Medical Sciences, No. 3 Yabao Road, Beijing, China; 3 Department of Thoracic Surgery, West China School of Medicine/West China Hospital of Sichuan University, No. 37 Guoxue Alley, Chengdu, 610041 Sichuan People’s Republic of China

**Keywords:** Esophageal cancer, Definitive chemoradiotherapy, Esophagostomy, Survival, Meta-analysis

## Abstract

**Background:**

We performed a meta-analysis to compare the efficacy of definitive chemoradiotherapy (dCRT) and esophagectomy as initial treatments for potentially resectable esophageal cancer.

**Methods:**

To assess both strategies, the combined odds ratios (ORs) and 95% confidence intervals (CIs) were calculated. Thirteen studies (*N* = 2071; dCRT = 869 and surgery = 1202) were included. In all, 90.39% of the patients were diagnosed with esophageal squamous cell carcinoma (ESCC).

**Results:**

The 2-year (OR = 1.199, 95% CI 0.922–1.560; *P* = 0.177) and 5-year overall survival (OS) rates (OR = 0.947, 95% CI 0.628–1.429; *P* = 0.796) were not significantly different. No significant differences were identified in the 2-year OS among patients with stage I disease (OR = 1.397, 95% CI 0.740–2.638; *P* = 0.303) or stage II–III (OR = 0.418, 95% CI 0.022–7.833; *P* = 0.560). Patients with lymph node metastases tended to have a better 5-year OS when treated with dCRT than with surgery (OR = 0.226, 95% CI 0.044–1.169; *P* = 0.076); however, the difference between the two methods was not significant. Western patients who received dCRT had poorer prognoses than patients who underwent surgery (OR = 1.522, 95% CI 1.035–2.238; *P* = 0.033). dCRT and surgery led to similar 5-year progression-free survival rates (OR = 1.06, 95% CI 0.79–1.42; *P* = 0.70).

**Conclusions:**

dCRT and surgery are equally effective as initial treatments for potentially resectable esophageal cancer. These results apply primarily to Asian populations as they have an increased incidence of ESCC.

**Electronic supplementary material:**

The online version of this article (10.1186/s12957-018-1470-y) contains supplementary material, which is available to authorized users.

## Background

Among all malignancies, esophageal cancer is the sixth most common cause of cancer-related death [1]. Esophageal squamous cell carcinoma (ESCC) is the dominant type of esophageal cancer in Asia [2]. While preoperative chemoradiotherapy can improve survival and local control [3, 4], surgery increases the risk of comorbidities and mortality, and patients who undergo surgery may experience a poor quality of life [5–8]. It has been reported that even in high-volume centres, surgery alone may lead to a 5% surgical mortality rate and a 10% mortality rate overall [9]. Furthermore, older patients are at a greater risk for surgical mortality following esophagectomy [10], and the safety and therapeutic effect of preoperative chemoradiation cannot be guaranteed in centres with little experience.

In clinical practice, surgery alone is frequently used as the primary treatment modality for esophageal cancer treatment modality, especially for less advanced esophageal tumours in patients in Asian countries [1]. One study showed that the rate of pathological complete response after chemoradiotherapy was 29% for all patients and was as high as 49% for ESCC patients [4]. Definitive chemoradiotherapy (dCRT) is used as the initial treatment in selected patients to avoid surgical mortality [11]. In patients with persistent or recurrent disease, salvage esophagectomy may be performed. Additionally, for stage I esophageal cancer patients in Japan, studies using chemoradiotherapy have demonstrated high rates of complete response and high survival rates with mild toxicity [12]. However, data on the comparative efficacies of dCRT and surgery are insufficient.

We therefore performed a meta-analysis to compare the therapeutic effects of dCRT and esophagectomy as initial treatments for resectable esophageal cancer. Subgroup analyses based on tumour stage, lymph node metastasis, and ethnicity were also conducted.

## Methods

### Search strategy

This study was conducted according to the Preferred Reporting Items for Systematic Reviews and Meta-Analyses (PRISMA) guidelines [13]. Two reviewers performed an independent systematic literature search. Databases were searched for studies as follows: PubMed (1985 to May 2016) and Web of Science (1992 to June 2018). The following search terms were used: (esophageal cancer or esophageal neoplasms) and (chemoradiotherapy or chemoradiotherapy) and (esophagectomy OR surgery).

### Inclusion and exclusion criteria

Studies were included if (1) they were randomised clinical trials (RCTs) or non-randomised clinical trials (nRCTs) that compared dCRT with surgery as the primary treatment in patients with resectable esophageal carcinoma, (2) they reported data on overall survival (OS) and progression-free survival (PFS) or if this information could be extracted from survival curves, and (3) the language of publication was English or Chinese. Studies that recruited patients who received neoadjuvant chemotherapy were excluded. Articles in which non-standardised scoring systems were used and those that reported insufficient data were also excluded.

### Data extraction

Each study was evaluated and classified by two independent investigators. Discrepancies were resolved by discussion and/or a third reviewer. The following data were extracted and listed: first author, year of publication, demographic characteristics, treatment regimen, OS, and PFS.

### Data analysis

This meta-analysis was conducted using STATA software version 12 (StataCorp, College Station, TX, USA). The primary endpoint was OS. We assessed and quantified statistical heterogeneity using Cochran’s *C* statistic and the *I*^2^ statistic. If heterogeneity was detected (*I*^2^ < 50% and *P* > 0.10), a fixed-effects model was adopted; otherwise, a random-effects model was used. A pooled analysis was performed with the combined odds ratio (OR) and 95% confidence intervals (CIs) using the *Z*-test. To assess potential publication bias, Begg’s test and Egger’s test were performed using STATA version 12. Data were considered statistically significant when *P* < 0.05.

## Results

### Characteristics of the studies

**Fig. 1:**
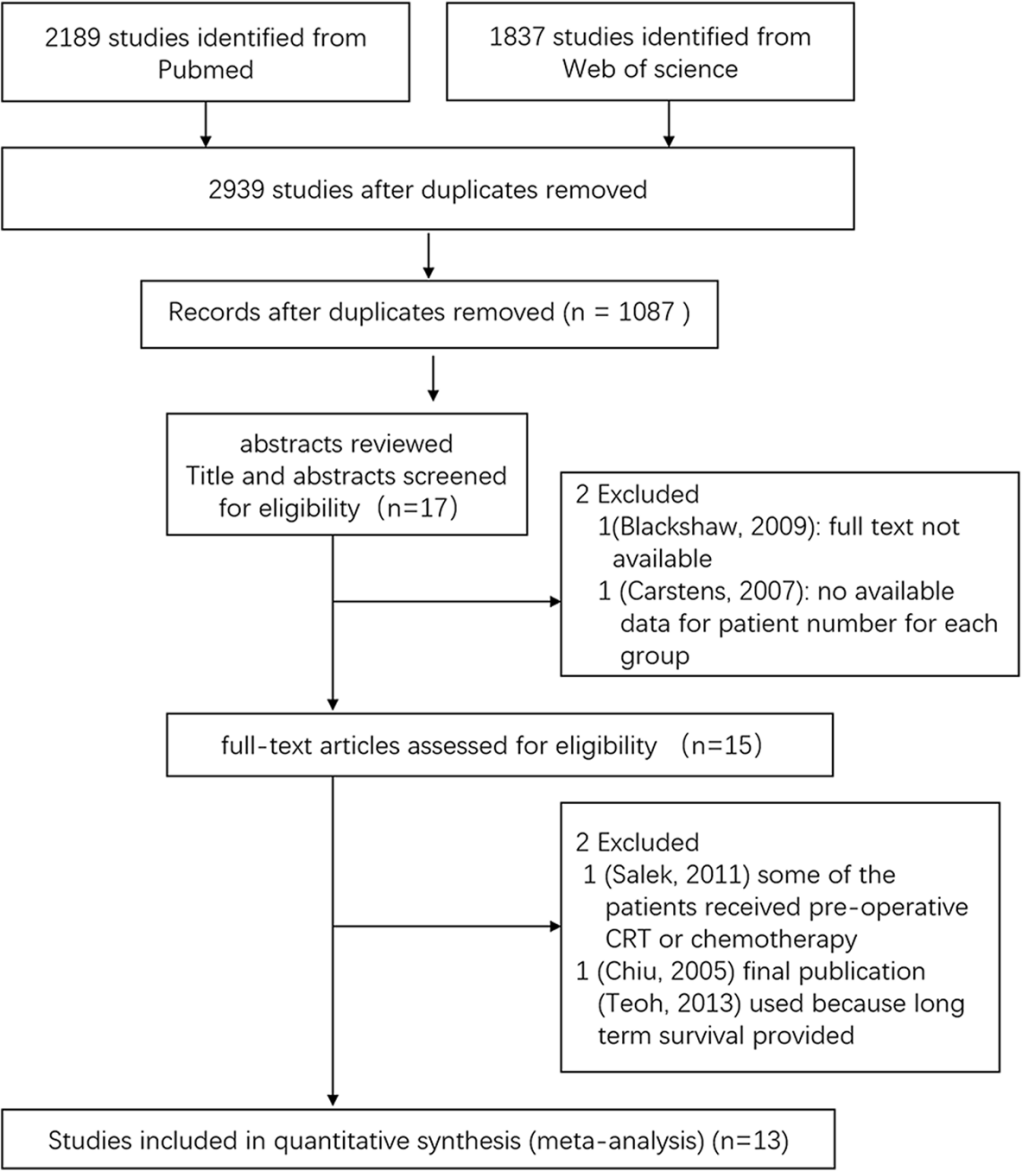
Flow diagram for article selection

The characteristics of the patient populations from all eligible studies are listed in Table 1. The selection process for eligible studies is shown in Fig. 1; we identified a total of 13 studies conducted between 1985 and 2015 that included 2071 patients and that compared dCRT (*N* = 869) with surgery (*N* = 1202). Of these 13 studies, 2 [14, 15] were randomised trials. The sample sizes ranged from 49 to 299 patients. Nine studies were restricted to patients with ESCC only, while 4 [16–19] enrolled patients with both ESCC and patients with adenocarcinoma; the predominant tumour histology of these 4 studies was ESCC (*N* = 1872 patients, 90.39%). Only 189 patients (9.13%) were diagnosed with adenocarcinoma, and 0.48% of the patients were diagnosed with cancer of other histological types. Overall, 712 (34%) patients had stage I disease. Most of the studies [14–16, 19–26] were performed in East Asia, including Korea, Japan, and China, while 2 studies [17, 18] were performed in Western countries.

**Table 1:** Characters and treatment regimens in trials included in the meta-analysis

Study	Study period	Country	Study design	Group	SCC, *n*(%)	EAC, *n*(%)	TN stage	Location	Treatment regimen	*N*	R0 rate %	Follow-up (months)
Chan 1999 [18]	1984–1994	Canada	nRCT	CRT	68(83)	14(17)	T1-3Nany	Thoracic/EGJ	RT 50–60 Gy concurrent with mitomycin C + 5-FU	82		U
				S	24(30)	57(70)			Transhiatal/thoracoabdominal esophagectomy	81	83	
Hironaka 2003 [22]	1992–1999	Japan	nRCT	CRT	53(100)	0	T2-3Nany	Thoracic	RT 60 Gy (2-week break) + PF (weekly, 5 weeks*2)	53		43
				S	45(100)	0			Total or subtotal thoracic esophagectomy with 3-field resection	45	98	
Sun 2006 [15]	1998–2002	China	RCT	CRT	134(100)	0	T1-3N0	Thoracic	RT (LCAF) 68.4–71 Gy	134		57
				S	135(100)	0			U	135		
Toh 2006 [21]	1995–2003	Japan	nRCT	CRT	25(100)	0	T1N0-1	Thoracic	RT 60 Gy + PF (5 days a week, 4 weeks)	25		32
				S	24(100)	0			Right transthoracic subtotal esophagectomy with 2/3-field dissection	24	88	
Yamashita 2009 [16]	2000–2009	Japan	nRCT	CRT	65(90)	5(7)*	T1N1 or T2-4N0-1	Cervical/thoracic	RT 50.4 Gy, 1.8 Gy/f, nedaplatin + 5-FU*4	72		37.8
				S	54(96)	0			Total/subtotal thoracic esophagectomy with at least 2-field lymphadenectomy.	56		
Yamashita 2008 [25]	2000–2005	Japan	nRCT	CRT	33(100)	0	T1-3Nany	Cervical/thoracic	RT 50.4 Gy + PF*2~4	33		36
				S	49(100)	0			Left thoracotomy by total or subtotal thoracic esophagectomy + least a 2-field lymphadenectomy	49	98	
Ariga 2009 [23]	2001–2005	Japan	nRCT	CRT	51(100)	0	T1-3N0-1	Thoracic	RT 60 Gy (including a 2-week break) + PF	51		49.7
				S	48(100)	0			Thoracoscopy + 2/3-field lymph node dissection.	48	91	36.4
Morgan 2009 [17]	1998–2005	UK	nRCT	CRT	93(53.8)	80(46.2)	T1-4Nany	Thoracic	RT 50 Gy, 2 Gy/f, PF*4	173		U
				S	18(14.3)	108(85.7)			2-phase method described by Lewis and Tanner.	126		
Yamamoto 2011 [26]	1995–2008	Japan	nRCT	CRT	54(100)	0	T1N0	Cervical/thoracic	RT 60 Gy concurrently with PF*2 cycles	54		30
				S	116(100)	0			Right thoracotomy + 2/3-field lymphadenectomy	116	100	67
Motoori 2012 [24]	1995–2007	Japan	nRCT	CRT	71(100)	0	T1bN0	Thoracic	RT ≥ 50 Gy concurrently with 5-FU and cisplatin-based chemotherapy	71		U
				S	102(100)	0			Subtotal esophagectomy via right thoracotomy with 2/3-field lymphadenectomy	102	100	
Teoh 2013 [14]	2000–2004	Hong Kong(China)	RCT	CRT	36(100)	0	T1-4N0-1	Mid/lower thoracic	RT 50–60 Gy, 2 Gy/f PF*3 weekly cycles	36		93
				S	44(100)	0			2- or 3-stage esophagectomy with 2-field lymphadenectomy	44	86.4	
Park 2014 [19]	2003–2012	Korea	nRCT	CRT	20(100)	0	T1N0	Thoracic	Induction XP + RT 54 Gy concurrently with XP/PF or RT alone	20		49
				S	256(97)	2(0.8)**			Ivor Lewis or McKeown, or a transhiatal esophagectomy, with 2/3-field lymph node dissection	264	98.9	
Matsuda 2015 [20]	2002–2011	Japan	nRCT	CRT	65(100)	0	T1-3N0-2	Thoracic	RT > 50 Gy + PF	65		46
				S	112(100)	0			Transthoracic esophagectomy with 2/3-field lymphadenectomy	112	87	

*RCT* randomised clinical trials, *nRCT* non-randomised clinical trials, *EGJ* esophagogastic junction, *RT* radiation therapy, *5-FU* 5-fluorouracil, *PF* fluorouracil and cisplatin, *LCAF* late course accelerated fractionation, *U* unavailable, *XP* cisplatin + capecitabine

*Two (3%) patients from dCRT group and three (4%) patients from surgery group had esophageal cancer with other pathological types other than ESCC or EAC

**Six (2.2%) patients from the surgery group had esophageal cancer with other pathological types other than ESCC or EAC

The radiotherapy dose, scheduling, and different chemotherapy regimens are presented in Table 1. All radiation treatments delivered in each study were definitive doses, and total doses ranged from 50 to 71.4 Gy. A platinum-based chemotherapy protocol was administered in most studies [14, 16, 17, 19–26]. The overall R0 resection rate, which was reported in 10 studies [14, 18–26], ranged from 83 to 100%.

### Effect of dCRT and surgery on OS

**Fig. 2:**
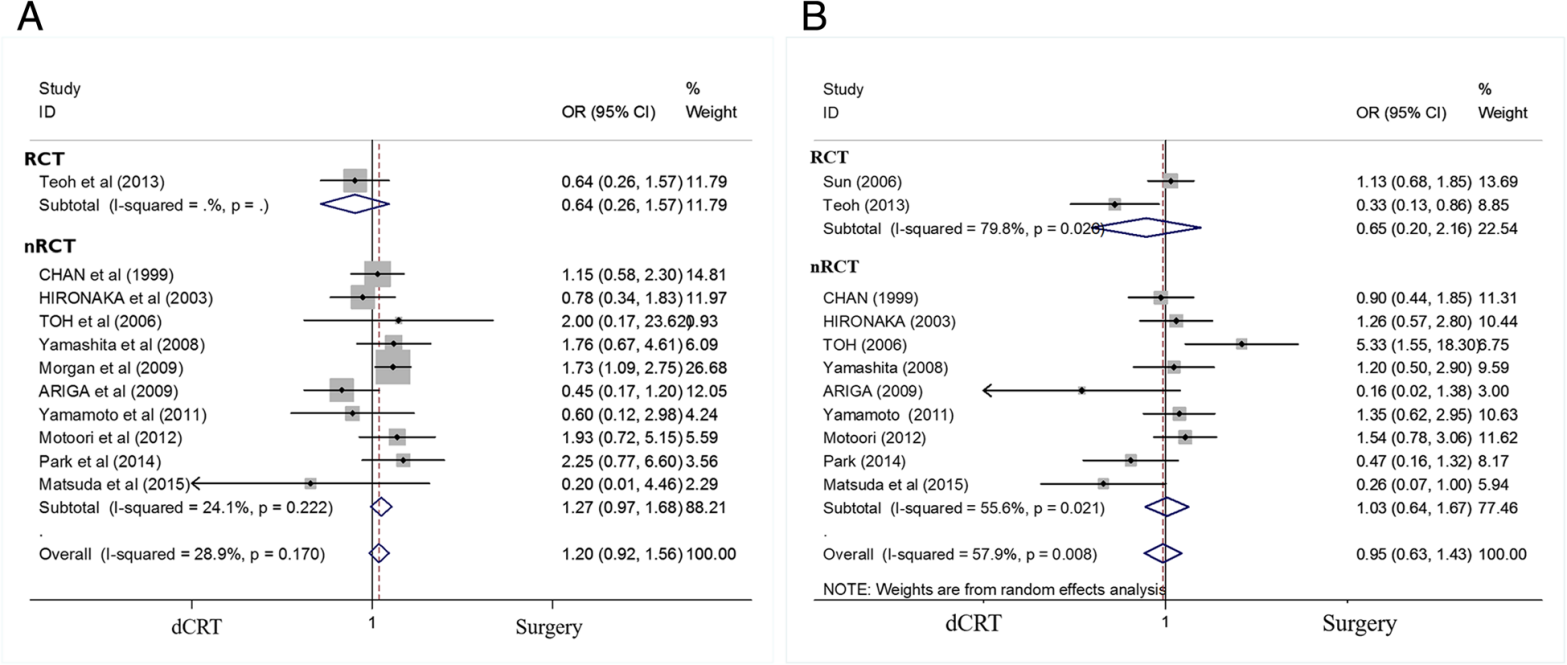
Forest plot comparison of the ORs of the OS between the dCRT and surgery arms. **a** The OR of the 2-year OS was 1.199 (95% CI 0.922–1.560; *P* = 0.177). Publication bias test: *P* = 0.640 (Begg’s test); *P* = 0.240 (Egger’s test). Weights are from fixed-effects analyses. **b** The OR of the 5-year OS was 0.947 (95% CI 0.628–1.429; *P* = 0.796). Publication bias test: *P* = 0.161 (Begg’s test), *P* = 0.236 (Egger’s test). Weights are from random-effects analyses

Figure 2 shows pooled estimates for OS in the randomised and non-randomised studies that compared dCRT with surgery. One study [16] was ineligible for the analysis of OS as only the PFS was reported. Both the short-term and long-term OS of patients treated with dCRT versus surgery were not significantly different. The pooled ORs for the 2-year and 5-year OS were 1.199 (95% CI 0.922–1.560; *P* = 0.177) and 0.947 (95% CI 0.628–1.429; *P* = 0.796), respectively.

### Effect of dCRT and surgery on the OS of patients with ESCC

**Fig. 3:**
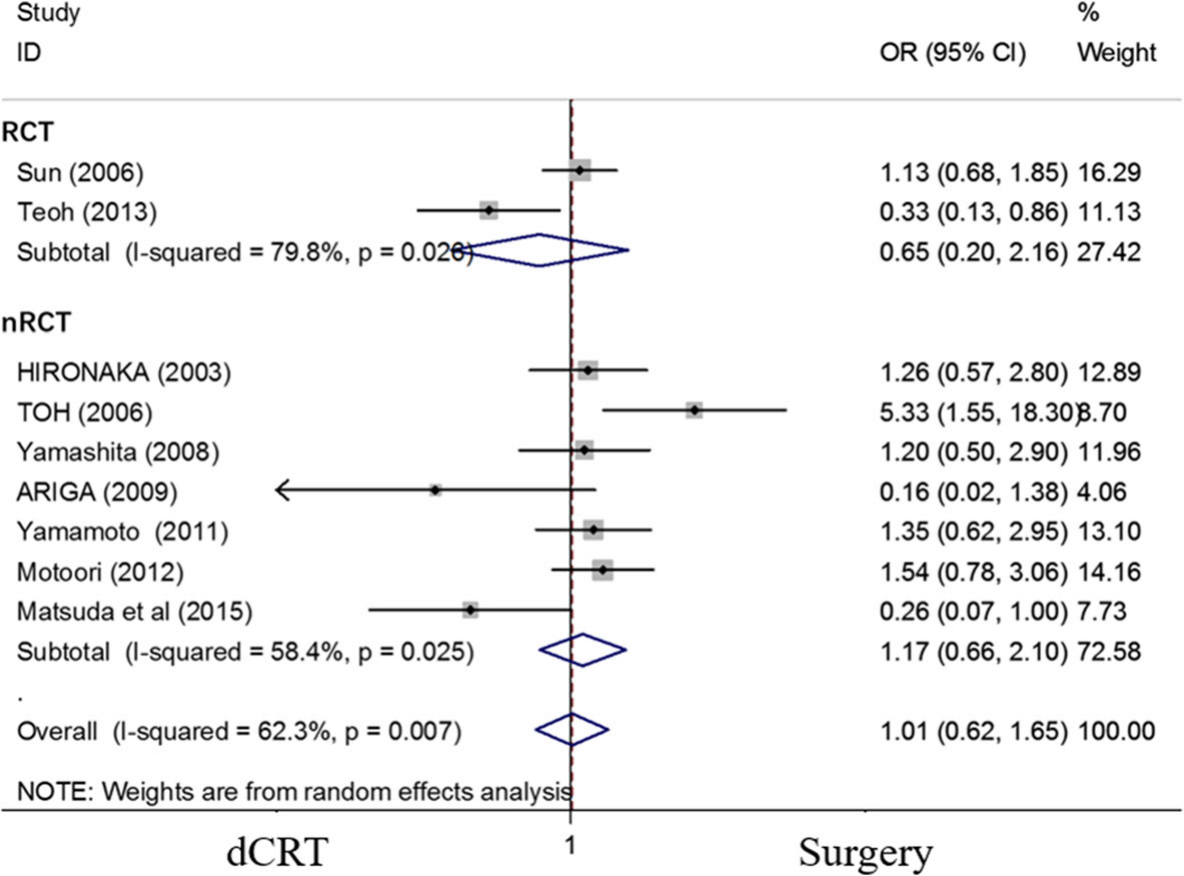
Forest plot comparison of the ORs of the OS between the dCRT and surgery arms for patients with ESCC. The OR of the 5-year OS was 1.015 (95% CI 0.623–1.652; *P* = 0.954). Publication bias test: *P* = 0.348 (Begg’s test), *P* = 0.350 (Egger’s test). Weights are from random-effects analyses

Nine studies [14, 15, 18, 20–26] were restricted to patients with ESCC. The pooled OR for the 5-year OS was not significantly different in patients with ESCC who were treated with dCRT compared with those who were treated with surgery (OR = 1.015, 95% CI 0.623–1.652; *P* = 0.954) (Fig. 3).

### Subgroup analyses of the effects of dCRT and surgery in patients with different stages of esophageal cancer

**Fig. 4:**
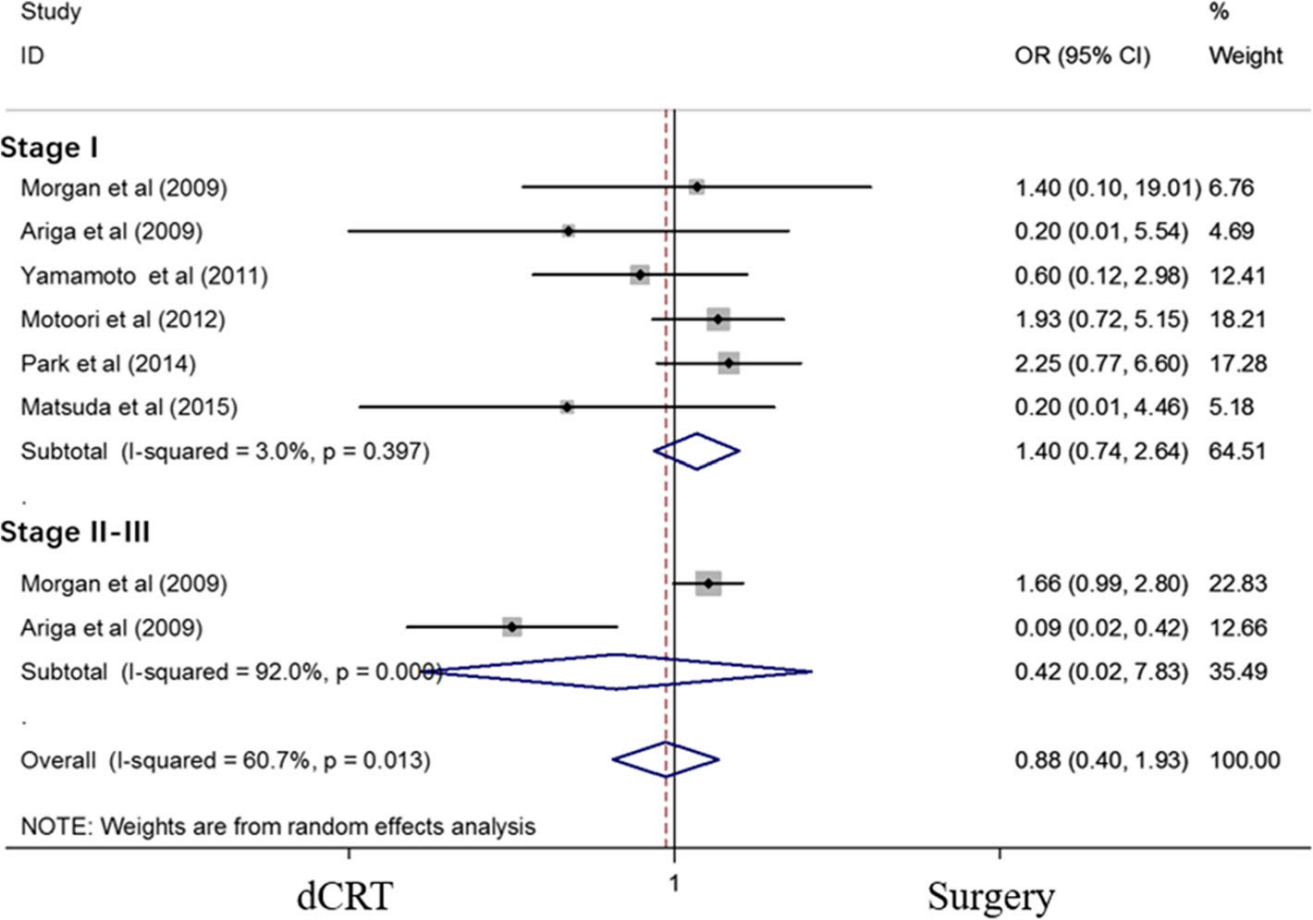
Forest plot comparison of the ORs of the OS between the dCRT and surgery arms for patients with different stages of esophageal cancer. The OR of the 2-year OS for stage I esophageal cancer was 1.397 (95% CI 0.740–2.638; *P* = 0.303). Publication bias test: *P* = 0.133 (Begg’s test), *P* = 0.039 (Egger’s test). The OR of the 2-year OS for stage II–III esophageal cancer was 0.418 (95% CI 0.022–7.833; *P* = 0.560). Publication bias (not available due to lack of studies). Weights are from random-effects analyses

Subgroup analyses of patients with stage I and stage II–III disease were performed, and none of the results demonstrated a significant difference between dCRT and surgery. The ORs for the 2-year OS of patients with stage I and stage II–III disease were 1.397 (95% CI 0.740–2.638; *P* = 0.303) and 0.418 (95% CI 0.022–7.833; *P* = 0.560), respectively (Fig. 4). An analysis of patients with stage I ESCC was also performed, and the OR of the 2-year OS was 1.021 (95% CI 0.488–2.134; *P* = 0.957) (Additional file 1: Figure S1).

### Subgroup analyses of patients with and without lymph node metastasis

**Fig. 5:**
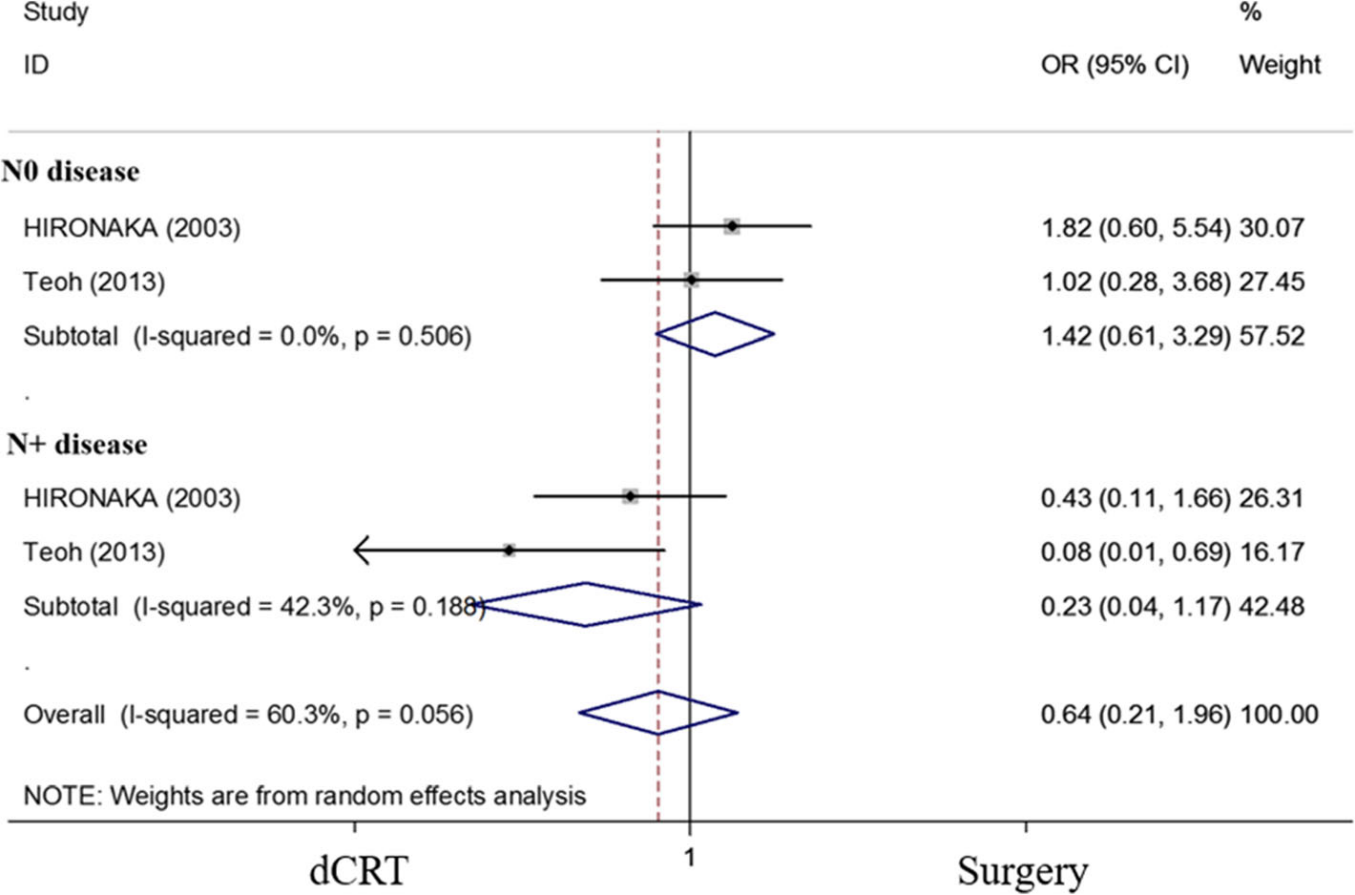
Forest plot comparison of the ORs of the OS between the dCRT and surgery arms for patients with N0 disease and N+ diseases. The OR of the 5-year OS for N0 disease was 1.419 (95% CI 0.613–3.289; *P* = 0.414). Publication bias: not available due to lack of studies. The OR of the 5-year OS for N+ disease was 0.226 (95% CI 0.044–1.169; *P* = 0.076). Publication bias: not available due to lack of studies. Weights are from random-effects analyses

We identified two studies [14, 22] that included data from patients with and without positive lymph nodes. In these studies, all enrolled patients were diagnosed with ESCC. A trend towards improved survival was observed in patients with positive lymph nodes who were treated with dCRT; however, the difference was not statistically significant (OR = 0.226, 95% CI 0.044–1.169; *P* = 0.076). For patients without lymph node metastasis, no significant difference was observed between the dCRT and surgery groups (OR = 1.419, 95% CI 0.613–3.289; *P* = 0.414) (Fig. 5). However, due to the small number of studies, heterogeneity was observed among patients with lymph node metastasis between the trials due to the small number of studies.

### Subgroup analyses of patients from Asian and Western countries

**Fig. 6:**
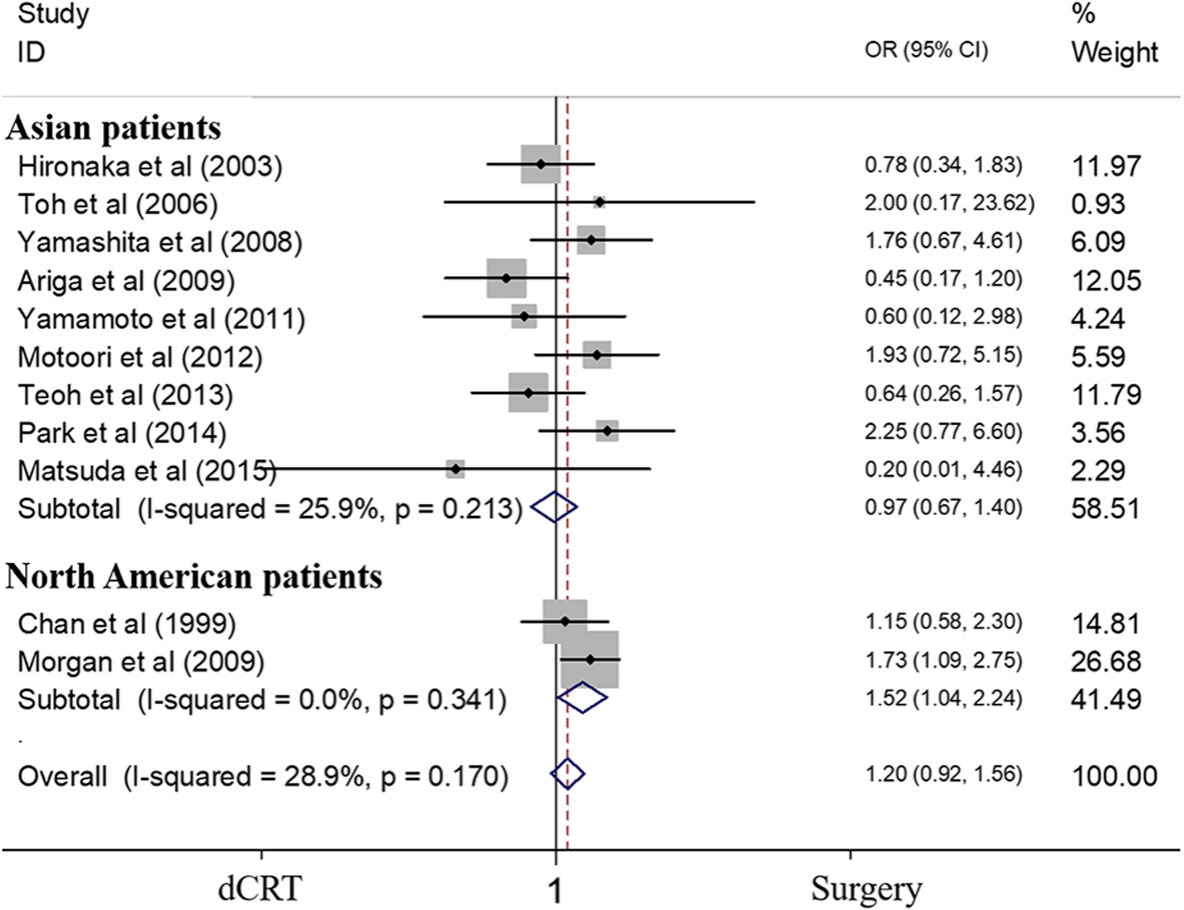
Forest plot comparison of ORs of the OS between the dCRT and surgery arms for Asian patients and Western patients. The OR of the 2-year OS for Asian patients was 0.970 (95% CI 0.674–1.395; *P* = 0.868). Publication bias test: *P* = 0.835 (Begg’s test); *P* = 0.807 (Egger’s test). The OR of the 2-year OS for Western patients was 1.522 (95% CI 1.035–2.238; *P* = 0.033). Publication bias: not available due to lack of studies. Weights are from fixed-effects analyses

We performed subgroup analyses to examine OS according to different regions. In this analysis, all patients from Asian countries were diagnosed with ESCC [14, 19–26], while Western studies included patients with esophageal adenocarcinoma (EAC) (56%) [17, 18]. The pooled results revealed no differences in terms of the 2-year OS of Asian patients who received dCRT compared with those who underwent surgery, while the estimated OR favoured surgery for patients from North America. The ORs for dCRT compared with that of surgery regarding the 2-year OS were 0.970 (95% CI 0.674–1.395; *P* = 0.868) and 1.522 (95% CI 1.035–2.238; *P* = 0.033) in Asian and Western patients, respectively (Fig. 6). We also performed an analysis on Asian patients with ESCC. The OR of the 2-year OS was 0.886 (95% CI 0.604–1.302; *P* = 0.538) (Additional file 2: Figure S2).

### Effect of dCRT and surgery on PFS

**Fig. 7:**
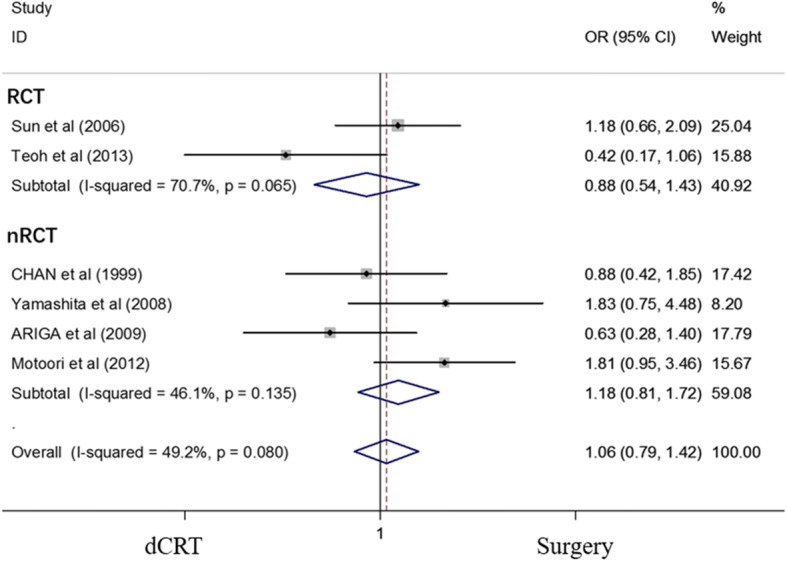
Forest plot comparison of ORs of the PFS between the dCRT and surgery arms. The OR of the 5-year PFS was 1.060 (95% CI 0.789–1.424; *P* = 0.698). Publication bias test: *P* = 0.260 (Begg’s test); *P* = 0.350 (Egger’s test). Weights are from fixed-effects analyses

Six studies [14, 15, 18, 23–25] reported the 5-year PFS. The results showed that dCRT is equivalent to surgery in terms of the 5-year PFS (OR = 1.06, 95% CI 0.79–1.42; *P* = 0.70) (Fig. 7). The 5-year PFS for ESCC patients between the dCRT and surgery arms was not significantly different. The OR of the 5-year PFS was 1.047 (95% CI 0.623–1.760; *P* = 0.862) (Additional file 3: Figure S3).

## Discussion

In this meta-analysis, the outcomes between dCRT and surgery as initial treatments for resectable esophageal cancer across 13 RCTs and nRCTs were compared. No statistically significant differences were observed in either short- or long-term OS or PFS. Subgroup analyses showed a trend towards improved outcomes for patients with positive lymph nodes who were treated with dCRT; however, the difference was not statistically significant. Patients from Western countries who underwent surgery had a better 2-year OS than those who received dCRT.

The number of clinical stage I esophageal cancer patients has recently increased [27, 28]. The survival rate following surgery for submucosal tumours is high; however, the postoperative quality of life is often compromised. Some studies [29, 30] have demonstrated encouraging clinical results for dCRT in these patients. In this meta-analysis, the 2-year OS of patients with stage I esophageal cancer was comparable between the dCRT and esophagostomy groups. Therefore, dCRT may be considered a treatment modality in selected patients. The ongoing JCOG0502 study by the Japan Clinical Oncology Group is investigating the non-inferiority of dCRT compared with surgery for stage I esophageal cancer patients.

Esophageal cancer is characterised by a high rate of lymph node metastasis [31], which is the most reliable predictor of survival after surgery [32]. In addition, because its pattern of spread is not always predictable and since skip node metastases may also occur, lymph node dissections may be difficult to perform. As suggested by our subgroup analyses, dCRT was superior to surgery among patients with lymph node metastases.

The pathological types of esophageal cancer are characterised by obvious demographic variations. The incidence of ESCC is much higher in Asia than in Western countries, whereas EAC accounts for only 1–4% of cases in Asian countries [2]. In addition, the incidence of EAC in Western countries is increasing rapidly [33]. We extracted data from all patients with ESCC and found no difference between dCRT and surgery in terms of long-term OS. Moreover, the subgroup analysis of the geographic areas showed that the 2-year OS was comparable between Asian patients who received dCRT and those who received surgery. In Western patients, surgical treatment has obvious therapeutic benefits. Studies on preoperative chemoradiotherapy [4, 6, 34] have shown that the pathological complete response rate of patients with EAC was lower than that of patients with ESCC. In this meta-analysis, two studies enrolled patients with EAC from Western countries [17, 18] (proportion of EAC, 44.1% and 62.9%), whereas almost all patients from Asian countries had ESCC. In addition, these two studies, which were performed in Western countries, included a large proportion of patients with lower esophageal cancer (66.9% and 77.3%). Patients with lower esophageal cancer were more amenable to surgery.

The progression rate of esophageal cancer is usually high when treated with either dCRT or surgery alone [34–37]. For long-term PFS, dCRT is equivalent to surgery when used as the initial treatment modality. A multidisciplinary approach is the ideal strategy, especially for the treatment of esophageal cancer.

This meta-analysis has several limitations. First, retrospective studies were included; therefore, selection bias may exist. For example, patients treated with dCRT in these studies were diagnosed with more advanced disease than those treated with surgery. Second, individual results from each patient were not applied. Third, modest heterogeneity was observed in terms of the surgical methods that were used and the dosing schedules between studies. In addition, the number of studies in the subgroup analyses was limited, especially those that included patients with lymph node metastasis and those with Western ethnicity. Finally, the studies were limited to two languages, which may present another bias.

## Conclusions

Our study demonstrates that dCRT is similar to surgery as an initial treatment for esophageal cancer with respect to the long-term survival of patients. Surgery may lead to a better OS in patients from Western countries, but further randomised trials are required to confirm these results.

## Additional files

Additional file 1: **Figure S1.** Forest plot comparison of ORs of the OS between the dCRT and surgery arms for stage I ESCC patients. The OR of the 2-year OS was 1.021 (95% CI 0.488–2.134; *P* = 0.957). Publication bias test: *P* = 0.308 (Begg’s test); *P* = 0.042 (Egger’s test). Weights are from fixed-effects analyses. (TIF 450 kb)

Additional file 2: **Figure S2.** Forest plot comparison of ORs of the OS between the dCRT and surgery arms for Asian ESCC patients. The OR of the 2-year OS was 0.886 (95% CI 0.604–1.302; *P* = 0.538). Publication bias test: *P* = 0.902 (Begg’s test); *P* = 0.769 (Egger’s test). Weights are from fixed-effects analyses. (TIF 764 kb)

Additional file 3: **Figure S3.** Forest plot comparison of ORs of the PFS between the dCRT and surgery arms for ESCC patients. The OR of the 5-year PFS was 1.047 (95% CI 0.623–1.760; *P* = 0.862). Publication bias test: *P* = 0.462 (Begg’s test); *P* = 0.432 (Egger’s test). Weights are from random-effects analyses. (TIF 601 kb)

## Abbreviations

5-FU: 5-Fluorouracil
CI: Confidence interval
dCRT: Definitive chemoradiotherapy
EAC: Esophageal adenocarcinoma
ESCC: Esophageal squamous cell carcinoma
LCAF: Late course accelerated fractionation
nRCT: Non-randomised clinical trial
OR: Odds ratio
OS: Overall survival
PF: Fluorouracil and cisplatin
PFS: Progression-free survival
PRISMA: Preferred Reporting Items for Reviews and Meta-Analyses
RCT: Randomised clinical trial
RT: Radiation therapy
U: Unavailable
XP: Cisplatin + capecitabine
