# Supplementary figures and images for: The role of definitive chemoradiotherapy versus surgery as initial treatments for potentially resectable esophageal carcinoma
Source: World J Surg Oncol. 2018 Aug 17;16:172. doi: 10.1186/s12957-018-1470-y (PMC6097217; doi:10.1186/s12957-018-1470-y)

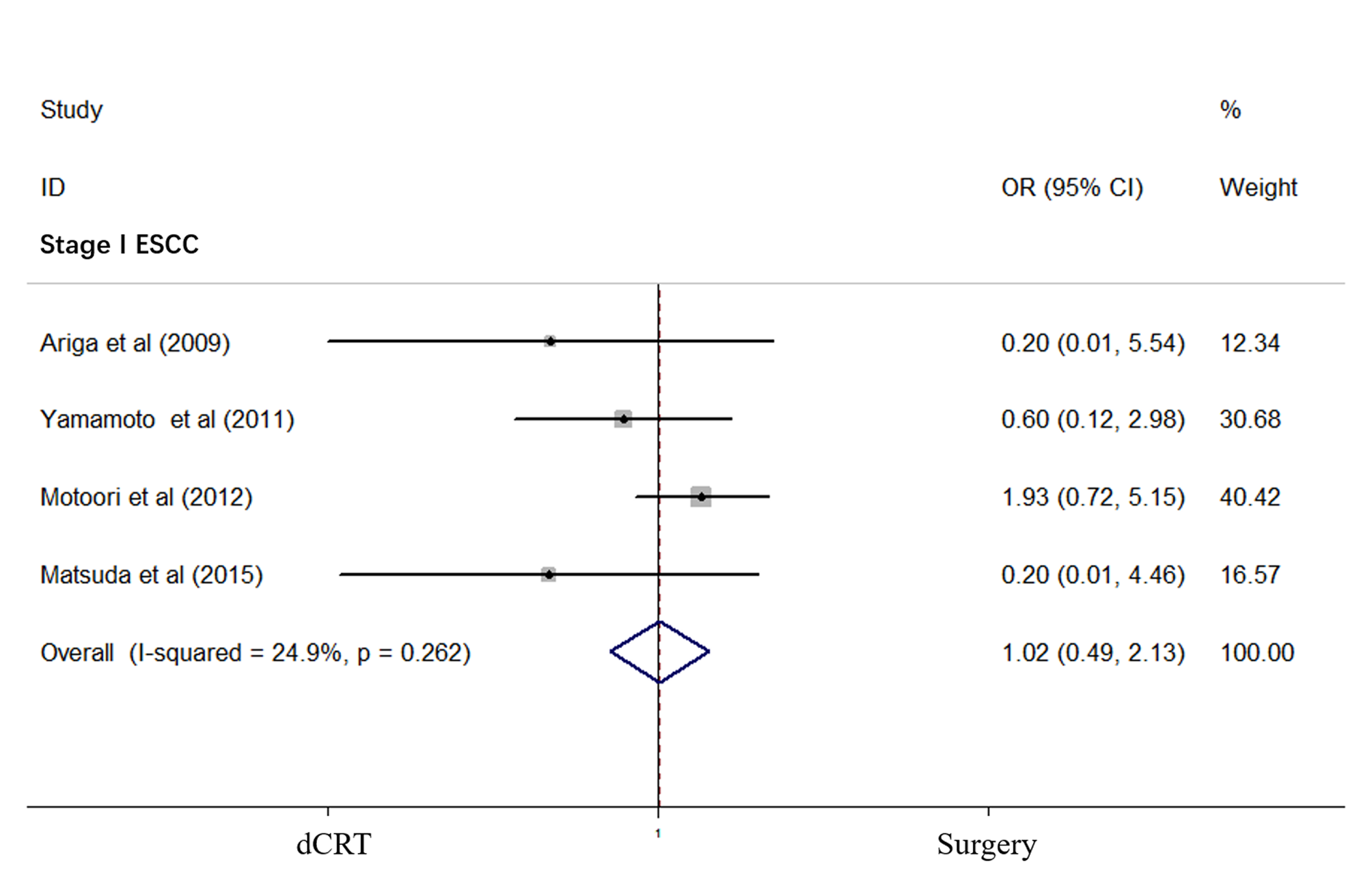

Supplement: Supplementary file 1 — Figure S1. Forest plot comparison of ORs of the OS between the dCRT and surgery arms for stage I ESCC patients. The OR of the 2-year OS was 1.021 (95% CI 0.488–2.134; P = 0.957). Publication bias test: P = 0.308 (Begg’s test); P = 0.042 (Egger’s test). Weights are from fixed-effects analyses. (TIF 450 kb) [file 12957_2018_1470_MOESM1_ESM.tif]

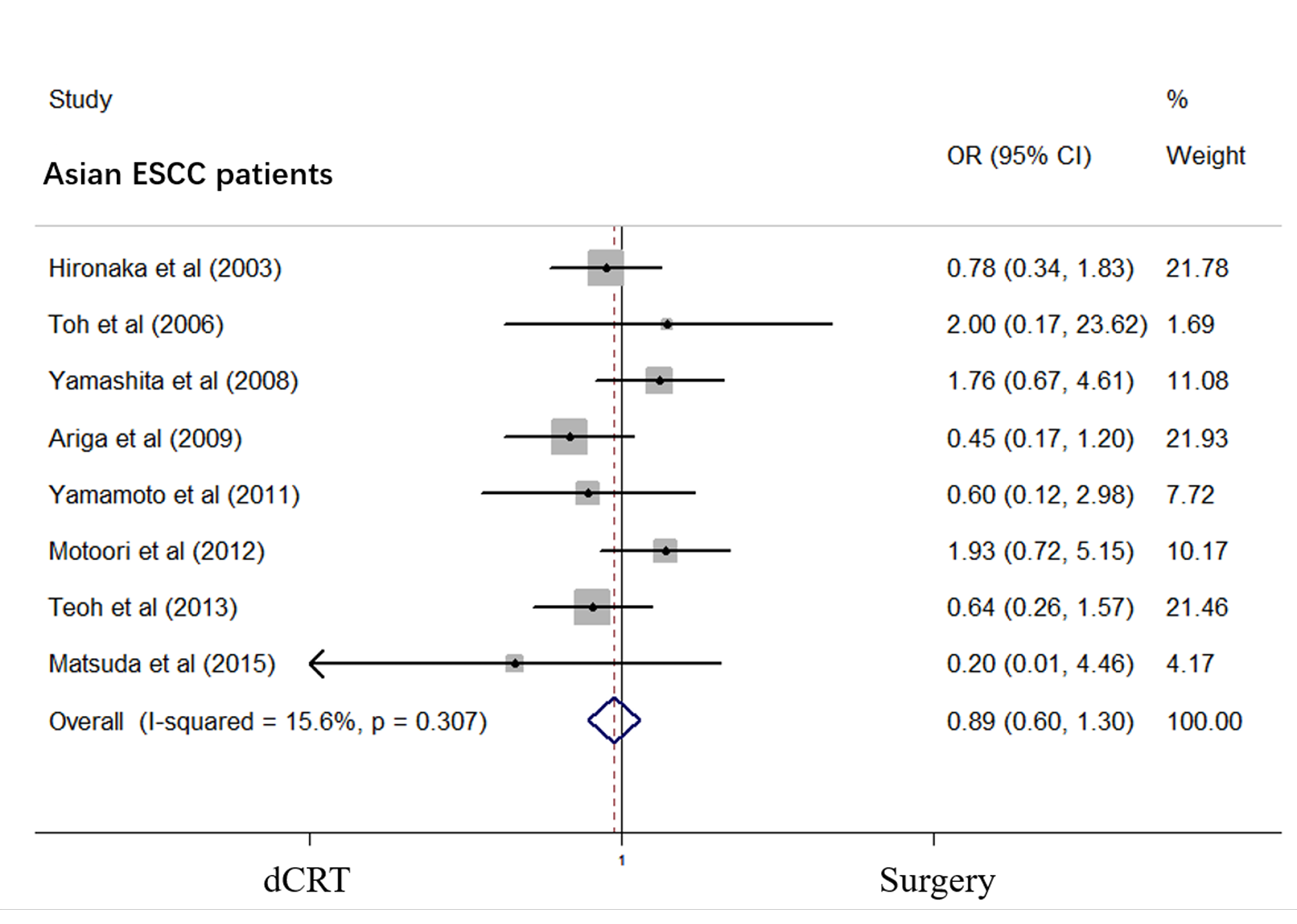

Supplement: Supplementary file 2 — Figure S2. Forest plot comparison of ORs of the OS between the dCRT and surgery arms for Asian ESCC patients. The OR of the 2-year OS was 0.886 (95% CI 0.604–1.302; P = 0.538). Publication bias test: P = 0.902 (Begg’s test); P = 0.769 (Egger’s test). Weights are from fixed-effects analyses. (TIF 764 kb) [file 12957_2018_1470_MOESM2_ESM.tif]

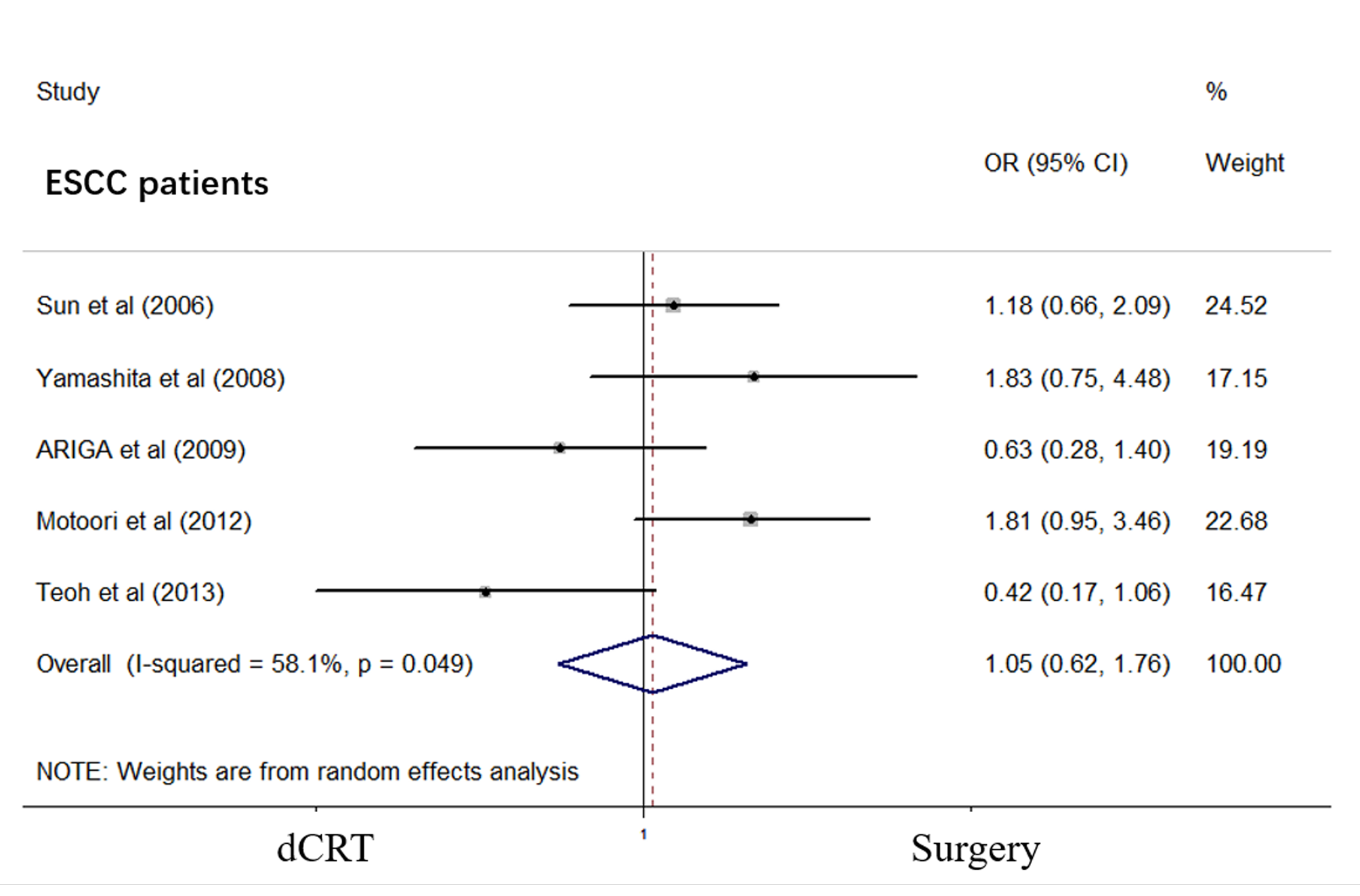

Supplement: Supplementary file 3 — Figure S3. Forest plot comparison of ORs of the PFS between the dCRT and surgery arms for ESCC patients. The OR of the 5-year PFS was 1.047 (95% CI 0.623–1.760; P = 0.862). Publication bias test: P = 0.462 (Begg’s test); P = 0.432 (Egger’s test). Weights are from random-effects analyses. (TIF 601 kb) [file 12957_2018_1470_MOESM3_ESM.tif]
